# Inhibitor of growth protein 4 interacts with Beclin 1 and represses autophagy

**DOI:** 10.18632/oncotarget.19033

**Published:** 2017-07-06

**Authors:** Valentina Sica, José Manuel Bravo-San Pedro, Guo Chen, Guillermo Mariño, Sylvie Lachkar, Valentina Izzo, Maria Chiara Maiuri, Mireia Niso-Santano, Guido Kroemer

**Affiliations:** ^1^ Université Paris Descartes, Sorbonne Paris Cité, Paris, France; ^2^ Equipe 11 labellisée Ligue Nationale contre le Cancer, Centre de Recherche des Cordeliers, Paris, France; ^3^ Institut National de la Santé et de la Recherche Médicale, Paris, France; ^4^ Université Pierre et Marie Curie, Paris, France; ^5^ Metabolomics and Cell Biology Platforms, Gustave Roussy Cancer Campus, Villejuif, France; ^6^ Departamento de Biología Fundamental, Instituto de Investigación Sanitaria del Principado de Asturias, Universidad de Oviedo, Spain; ^7^ Centro de Investigación Biomédica en Red en Enfermedades Neurodegenerativas, Cáceres, Spain; ^8^ Facultad de Enfermería y Terapia Ocupacional, Universidad de Extremadura, C.P, Cáceres, Cáceres, Spain; ^9^ Pôle de Biologie, Hôpital Européen Georges Pompidou, AP-HP, Paris, France; ^10^ Department of Women's and Children's Health, Karolinska University Hospital, Stockholm, Sweden

**Keywords:** cancer, ING4, PIK3C3, TP53, Autophagy

## Abstract

Beclin 1 (BECN1) is a multifunctional protein that activates the pro-autophagic class III phosphatidylinositol 3-kinase (PIK3C3, best known as VPS34), yet also interacts with multiple negative regulators. Here we report that BECN1 interacts with inhibitor of growth family member 4 (ING4), a tumor suppressor protein that is best known for its capacity to interact with the tumor suppressor protein p53 (TP53) and the acetyltransferase E1A binding protein p300 (EP300). Removal of TP53 or EP300 did not affect the BECN1/ING4 interaction, which however was lost upon culture of cells in autophagy-inducing, nutrient free conditions. Depletion of ING4 stimulated the enzymatic activity of PIK3C3, as visualized by means of a red fluorescent protein-tagged short peptide (FYVE) that specifically binds to phosphatidylinositol-3-phosphate (PI3P)-containing subcellular vesicles and enhanced autophagy, as indicated by an enhanced lipidation of microtubule-associated proteins 1A/1B light chain 3 beta (LC3B) and the redistribution of a green-fluorescent protein (GFP)-LC3B fusion protein to cytoplasmic puncta. The generation of GFP-LC3B puncta stimulated by ING4 depletion was reduced by simultaneous depletion, or pharmacological inhibition, of PIK3C3/VPS34. In conclusion, ING4 acts as a negative regulator of the lipid kinase activity of the BECN1 complex, and starvation-induced autophagy is accompanied by the dissociation of the ING4/BECN1 interaction.

## INTRODUCTION

Macroautophagy, to which we refer as ‘autophagy’, is a catabolic process that cells use to cope with stressful conditions such as nutrient deprivation, organelle dysfunction or invasion of the cytoplasm by infectious pathogens [1, 2]. Alterations in autophagy are linked to multiple diseases, including aging [3], cancer [4], and neurodegenerative disorders [5]. Autophagy consists in the sequestration of cytoplasmic material in double-membraned vesicles (autophagosomes) that fuse with lysosome (autophagolysosomes) where the luminal content is degraded by the action of hydrolases operating at low pH [6].

The formation of autophagosomes is finely regulated by the concerted action of protein kinases, in particular unc-51 like autophagy activating kinases (ULK1/ULK2), the beclin 1 (BECN1) lipid kinase complex and an ubiquitin-like conjugation system [7]. The first step of vesicles nucleation depends on the activation of phosphatidylinositol 3-kinase catalytic subunit type 3 (PIK3C3, best known as VPS34), which operates in the context of the BECN1 complex [8]. Numerous additional proteins interact with BECN1 to activate or inhibit VPS34 and hence to initiate or suppress autophagy [9–12].

The evolutionarily conserved inhibitor of growth family (ING) [13] consists of several proteins that are generally considered as tumor suppressors and possess a similar domain organization [14]. ING proteins are highly homologous in the C terminal domain. Each of them harbors a plant homeodomain (PHD), a nuclear localization signal (NLS), as well as a noncoding region (NCR) [13]. The five proteins from the ING family (ING1-5) are involved in the regulation of cell cycle progression, apoptosis, senescence and DNA repair [15, 16]. Importantly, all ING proteins modulate the enzymatic (de)acetylase activity of histone acetyl transferases (HATs) and histone deacetylases (HDACs), thereby influencing histone acetylation and gene transcription [17, 18].

In several types of malignancy, the expression of ING proteins is reduced [19]. Ovarian, breast, head & neck carcinomas, as well as melanomas exhibit decreased expression of *ING1* [20]. Lung cancer and melanoma often show reduced levels of ING2 [21, 22], while gliomas, advanced breast cancers and cervical and uroepithelial carcinomas underexpress *ING4* [23–26]. ING4 loss is also an unfavorable prognostic marker in colorectal cancer [27] and gastrointestinal stromal tumors [28]. For this reason, studies have been launched to (re)express ING4 in cancers by means of viral vectors, with some encouraging preclinical results [29–33].

ING4 can arrest the cell cycle of HepG2 hepatocarcinoma cells and induce apoptosis by means of activation of the tumor suppressor p53 (TP53) and subsequent upregulation of cyclin-dependent kinase inhibitor 1 (CDK1, best known as p21) [34]. ING4 participates to a chromatin-binding multiprotein complex, together with TP53 and acetyltransferase E1A Binding Protein P300 (EP300), in which TP53 is acetylated by EP300, resulting in its transcriptional activation [35]. Moreover, ING4 binds to nuclear factor NFκB p65 subunit (encoded by the *RelA* gene), targeting it for ubiquitinylation and proteasomal destruction [36], meaning that ING4 deficiency can unleash NFκB activation and favor angiogenesis in glioblastoma [23]. Moreover, the PHD domain of ING4 directly binds to histone H3 trimethylated at lysine 4 (H3K4me3), thereby recruiting the HBO1 histone HAT complex to target promoters, thereby facilitating histone H3 acetylation [37, 38].

Our group published the results of a yeast-2-hybrid screen suggesting that ING4 is part of the BECN1 interactome [12]. Intrigued by this observation, we investigated the putative role of ING4 as an autophagy regulator. Here we report evidence that ING4 indeed interacts with BECN1 in baseline conditions, in human cells. Importantly, nutrient depletion, which is (one of) the most physiological inducer(s) of autophagy [39], causes ING4 to dissociate from BECN1. Moreover, depletion of ING4 was sufficient to increase autophagic flux, supporting the idea that ING4 acts as a potent endogenous inhibitor of autophagy.

## RESULTS AND DISCUSSION

**Figure 1:**
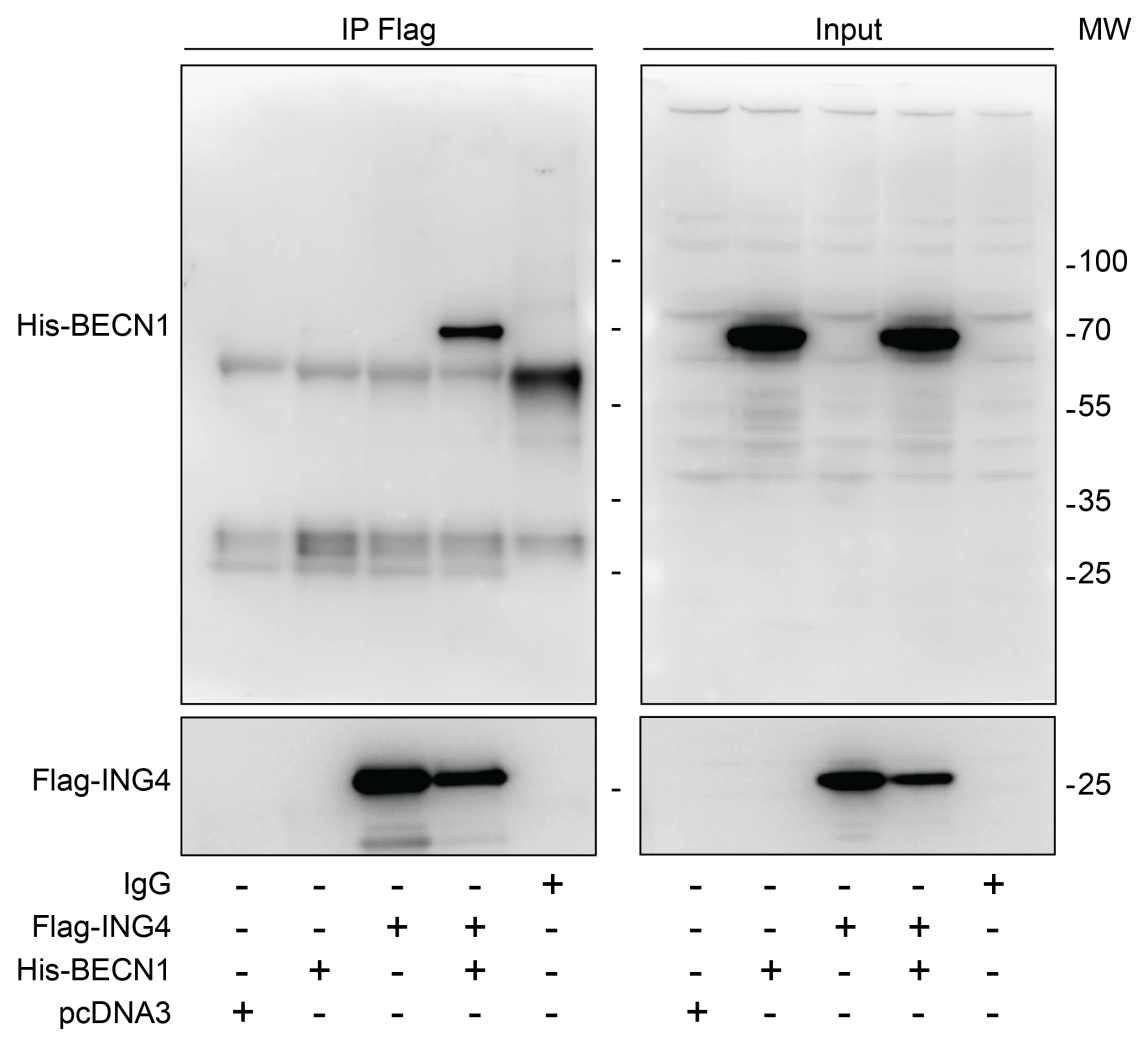
Interaction between ING4 and BECN1 Co-immunoprecipitation of BECN1 with ING4. The indicated constructs, namely Flag-tagged ING4 (Flag-ING4) and His-tagged BECN1 (His-BECN1) were transfected into U2OS cells alone or in combination. The pcDNA3 construct was transfected as internal control of the experiment. Forty-eight hours later, ING4 was immunoprecipitated with a specific antibody for Flag and the precipitate was separated by SDS-PAGE and revealed with an antibody specific for His (upper panels) or Flag (down panels). Results are representative of three independent experiments. Immunoprecipitation (IP), molecular weight (MW).

**Figure 2:**
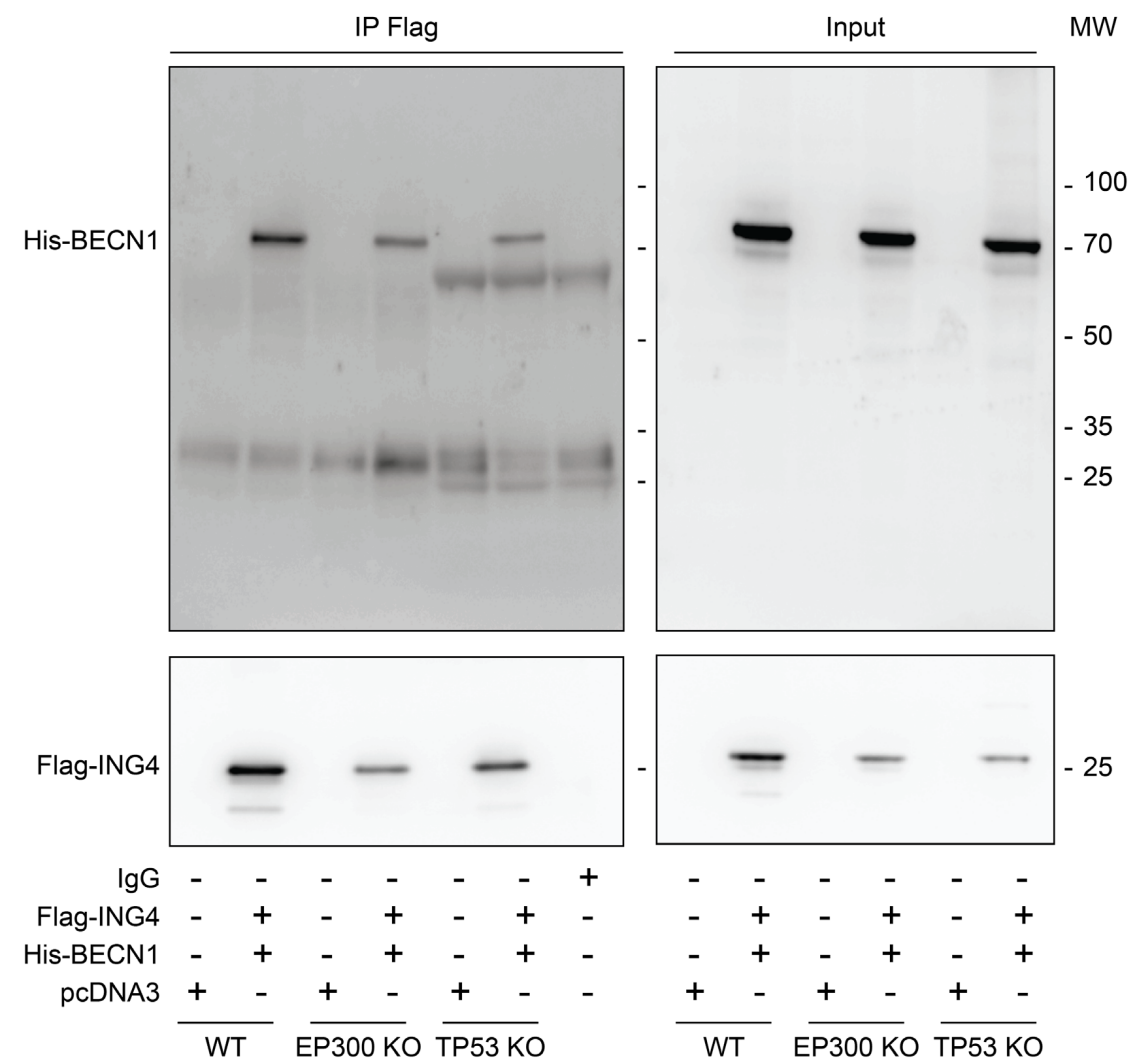
EP300 or TP53 are dispensable for the interaction between ING4 and BECN1 Co-immunoprecipitation of BECN1 with ING4. The indicated constructs, namely Flag-tagged ING4 (Flag-ING4) and His-tagged BECN1 (His-BECN1) were co transfected into HCT116 wild-type (WT), EP300 or TP53 knock-out (KO) cells. The pcDNA3 construct was transfected as internal control of the experiment. Forty-eight hours later, ING4 was immunoprecipitated with a specific antibody for Flag and the precipitate was separated by SDS-PAGE and revealed with a specific antibody for His and also with a specific antibody for Flag as control. Results are representative of three independent experiments. Immunoprecipitation (IP), molecular weight (MW).

Interaction between ING4 and BECN1. Previous work has revealed that the N-terminal domain of ING4 (4-150 residues among 245) interacts with human BECN1 protein in a yeast-two-hybrid system [12]. To investigate whether this interaction occurs in human cells, we transfected human osteosarcoma U2OS cells with Flag-tagged ING4 alone or together with His-tagged BECN1. Upon pull-down of Flag-ING4, His-BECN1 was detectable in the immunoprecipitate (Figure 1). While the immunoprecipitate of Flag-ING4 contained multiple acetylated proteins, there was no particular increase in protein acetylation of the co-transfected His-BECN1 (approximate molecular weight 70 kDa), suggesting that BECN1 itself is not a target of the acetyltransferase activity of the complex in which ING4 takes part (Supplemental Figure 1S). Indeed, ING4 is known to interact with the acetyltransferase EP300 and the tumor suppressor TP53 [35]. We therefore investigated whether the interaction between Flag-ING4 and His-BECN1 would depend on EP300 or TP53. However, the immunoprecipitate of Flag-ING4 continued to contain His-BECN1 in human colorectal HCT116 cells in which EP300 or TP53 were removed by homologous recombination [40] (Figure 2). Altogether, these data plead in favor of a direct protein-protein interaction between ING4 and BECN1, confirming the yeast-2-hybrid data [12].

**Figure 3:**
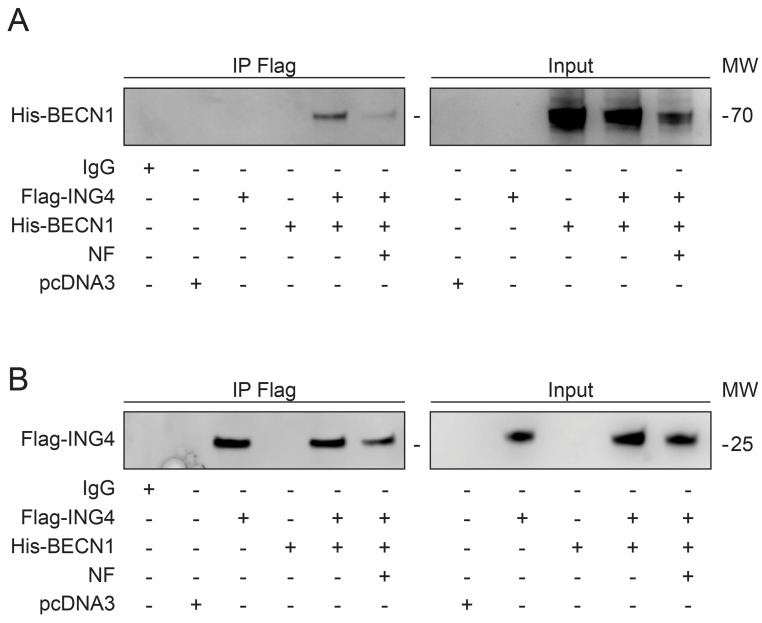
Starvation induces autophagy and reduces the association of BECN1-ING4 in U2OS cells (**A**, **B**) The indicated constructs, namely Flag-tagged ING4 (Flag-ING4) and His-tagged BECN1 (His-BECN1) were transfected, alone or in combination, into U2OS cells. The pcDNA3 construct was transfected as internal control of the experiment. Forty-eight hours later, cells were treated with complete or nutrient free (NF) medium for 6h and consequently, ING4 was immunoprecipitated with a specific antibody for Flag. The precipitate was separated by SDS-PAGE and revealed with specific antibodies for His (A) and Flag (B). Immunoprecipitation (IP), molecular weight (MW).

Reduction of the interaction between ING4 and BECN1 upon starvation. The most physiological stimulus for autophagy induction is starvation [39, 41, 42]. When U2OS cells were cultured in nutrient-free (NF) conditions, the interaction between Flag-ING4 and His-BECN1 was largely reduced (Figure 3A) although starvation barely affected the expression levels of Flag-ING4 (Figure 3B). These findings suggest that the physiological induction of autophagy correlates with alterations in the BECN1 interactome that include a reduction in the interaction with ING4.

**Figure 4:**
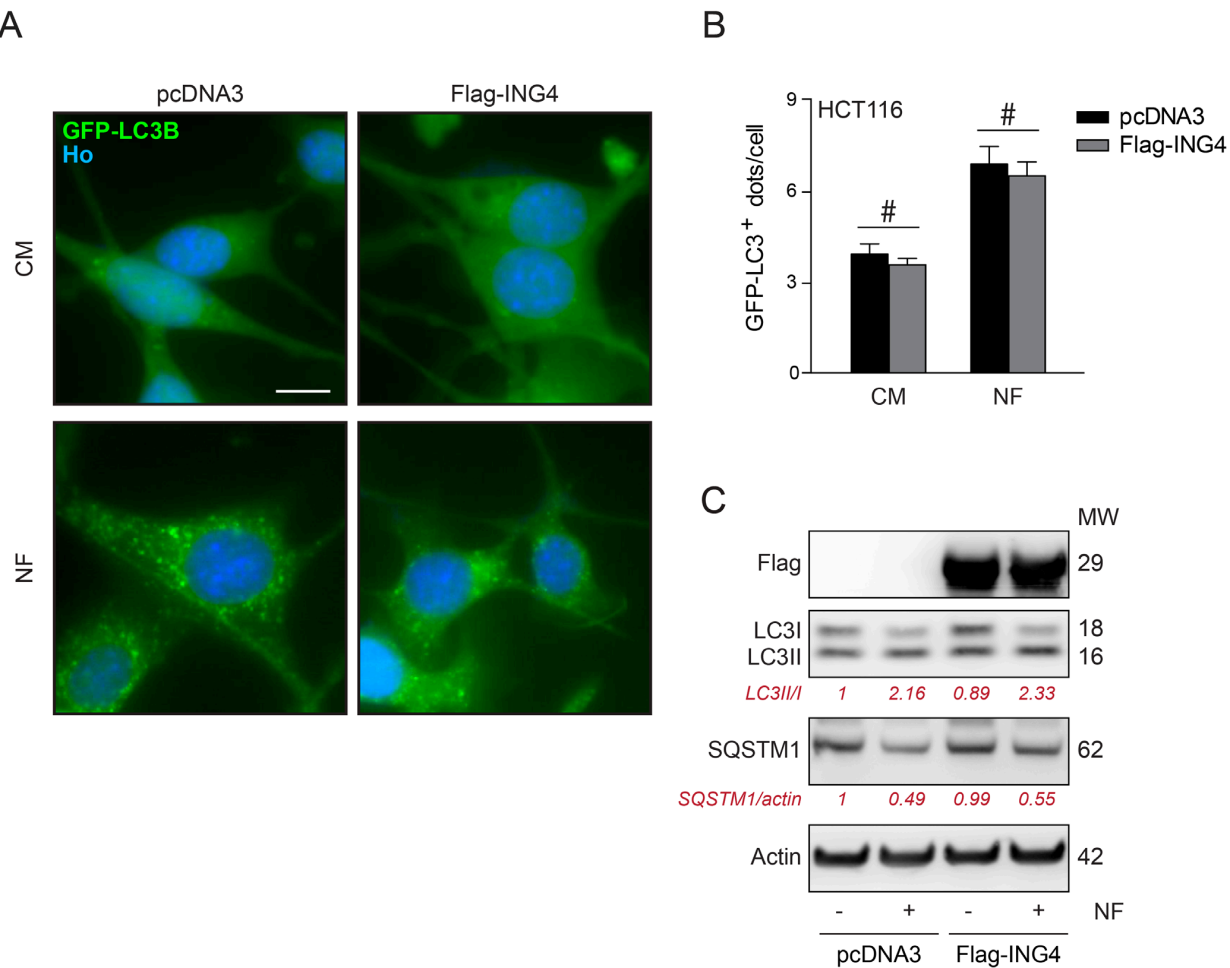
Overexpression of ING4 has not inhibitory effect on starvation induced autophagy **A.** Representative photomicrographs of empty vector (pcDNA3) and ING4 (Flag-ING4) overexpressing HCT116 cells stably expressing GFP-LC3 treated in presence of complete (CM) or nutrient free (NF) medium for 6h. Hoechst 33342 (Ho, blue) represents nuclear staining. GFP-LC3B puncta (green) correspond to autophagosomes. Scale bars: 10 μm. **B.** Quantification of GFP-LC3B puncta in cells treated in CM or NF medium. Data are means ± SD (*n* = 5). Statistical analysis was performed by Student's t test in comparison as indicated, # *p* > 0.05, not significant. **C.** Western blot detection and quantification of LC3 lipidation and SQSTM1 degradation in ING4 overexpressing HCT116 cells upon starvation (6h). Actin was used as a loading control. Densitometry was employed to quantify the abundance of lipidated LC3 (LC3-II/LC3I ratio) and SQSTM1 (normalized to actin levels). Results are representative of three independent experiments. Molecular weight (MW).

Inhibition of autophagy by ING4. Overexpression of Flag-ING4 had no major effect on the frequency of GFP-LC3-positive puncta in the cytoplasm of HCT116 and U2OS cells, and failed to cause a major reduction in autophagic puncta generated in response to NF conditions (Figure 4A, 4B) or autophagy enhancers as Torin1 or rapamycin (Rapa) respectively (Supplemental Figure 2S). A similar marginal effect could be seen when the ratio of lipidated (electrophoretically more mobile) over non-lipidated (less mobile) LC3 and SQSTM1 (best known as p62) degradation were determined by immunoblot as a surrogate of the membrane distribution of LC3 (Figure 4C). This suggests that overexpression of ING4 is unable to inhibit autophagy induced by starvation.

**Figure 5:**
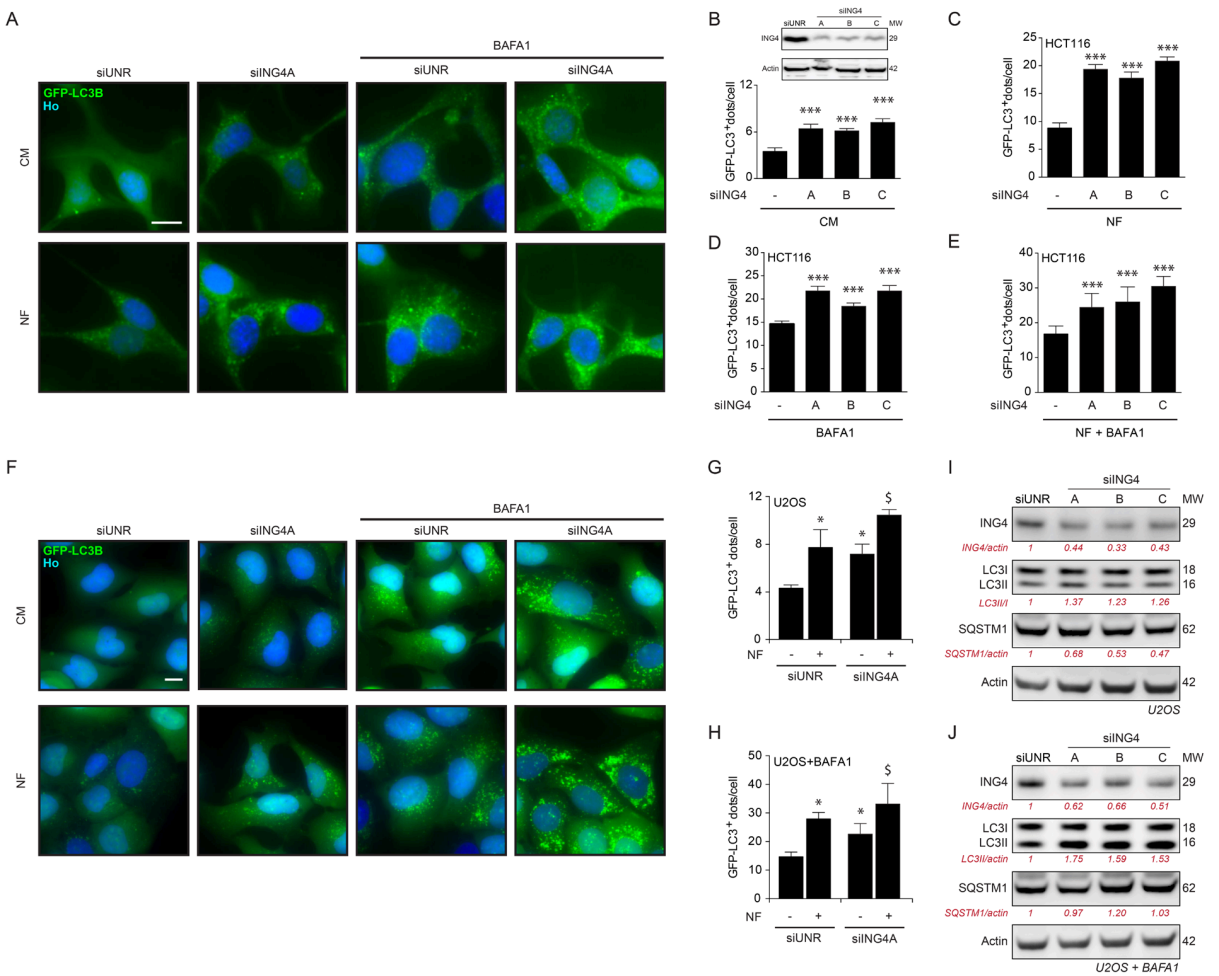
ING4 silencing induces autophagy **A.** Representative photomicrographs of ING4 knockdown (siING4A) HCT116 cells stably expressing GFP-LC3 treated with complete (CM) or nutrient free (NF) medium for 6h, in presence or absence of 1μM bafilomycin A1 (BAFA1) for the last 3h. The non-targeting siRNA (siUNR) was used as internal control of the experiments. Hoechst 33342 (Ho, blue) represents nuclear staining. GFP-LC3B puncta (green) correspond to autophagosomes. Scale bars: 10 μm. **B.**-**E.** Quantification of GFP-LC3B dots in ING4 knockdown HCT116 cells treated in CM or NF medium for 6h in presence or absence of BAFA1 for the last 3h. To knockdown ING4, 3 different siRNAs were used (siING4A, siING4B and siING4C). Western blot proves the efficiency of siING4s. Data are means ± SD (*n* = 5). Statistical analysis was performed by Student's t test in comparison with the control condition, ***p < 0.001. **F.** Representative photomicrographs of siING4A knockdown U2OS cells stably expressing GFP-LC3, treated with NF medium for 6h or CM, in presence or absence of 1 μM BAFA1 for the last 3h. Hoechst 33342 (Ho, blue) represents nuclear staining. GFP-LC3B puncta (green) correspond to autophagosomes. Scale bars: 10 μm. **G.**, **H.** Quantification of GFP-LC3B dots in siING4A U2OS cells treated in the same conditions described in (F). Data are means ± SD (*n* = 5). Statistical analysis was performed by Student's t test in comparison with the control condition (siUNR, *p < 0.05 ; siING4A, $ *p* < 0.05). **I.**, **J.** Western blot detection of ING4 levels, LC3B conversion and SQSTM1 degradation in U2OS knockdown cells with 3 different ING4 siRNAs in absence (I) or presence (J) of 1 μM BAFA1 for 3h. Actin was used as a loading control. Densitometry was employed to quantify the efficiency of the ING4 silencing and abundance of lipidated LC3 (LC3-II/LC3 I ratio or LC3II/actin ratio when BAFA1 is added) and SQSTM1 (normalized to actin levels). Results are representative of three independent experiments. Molecular weight (MW).

Next, we depleted ING4 using three distinct, non-overlapping small interfering RNAs (siRNAs). All siRNAs caused an increase in the number of GFP-LC3B-positive puncta in HCT116 cells, both in fed conditions (Figure 5A, 5B) as well as in conditions of starvation, in which the depletion of ING4 caused a hyperinduction of autophagic puncta (Figure 5A, 5C). In the presence of bafilomycin A1 (BAFA1) for 3h, which inhibits the fusion of lysosomes with autophagosomes, thus blocking the last step of autophagy, the increase in GFP-LC3-positive puncta by ING4 depletion was also detectable, both in baseline conditions (Figure 5A, 5D) and upon starvation (Figure 5A, 5E). The same results were also observed in U2OS cells (Figure 5F-5H). Upon ING4 depletion, such cells also manifested a shift in the ratio between lipidated and non-lipidated LC3 that is compatible with an induction of autophagy, in baseline condition (Figure 5I) or in presence of BAFA1 for 3h (Figure 5J). Altogether, these data demonstrate that endogenous ING4 can act as repressor of autophagy.

**Figure 6:**
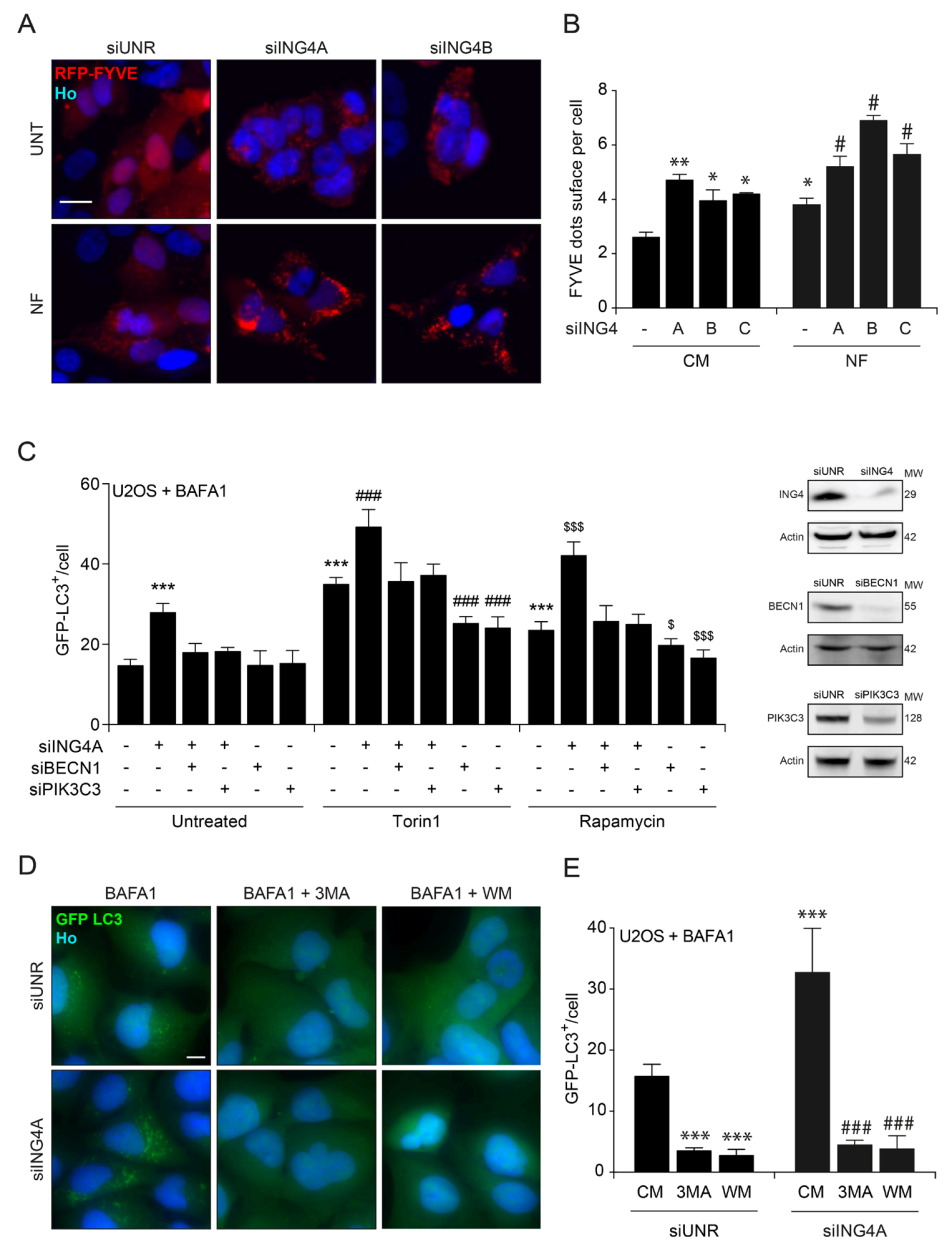
ING4 silencing leads to autophagy due to an increase of the PIK3C3 activity **A.** Representative photomicrographs of ING4 knockdown (siING4A, siING4B) U2OS cells stably expressing RFP-tagged short peptide (FYVE) treated with complete (CM) or nutrient free (NF) medium for 6h. The non-targeting siRNA (siUNR) was used as internal control of the experiments. Hoechst 33342 (Ho, blue) represents nuclear staining. RFP-tagged short peptide (red) corresponds to activation of PIK3C3. Scale bars: 10 μm. **B.** Quantification of FYVE puncta upon ING4 silencing (siING4A, siING4B, siING4C), in cells treated with CM or NF for 6h. Data are means ± SD (*n* = 5). Statistical analysis was performed by Student's t test in comparison with the control condition (**p < 0.01, *p < 0.05) or in comparison with the NF condition (# *p* < 0.05). **C.** Quantification of the number of GFP-LC3B dots in U2OS cells knockdown for ING4 (siING4A), Beclin 1 (siBECN1) or PIK3C3 (siPIK3C3) alone or in combination as indicated. Western blot proves the efficiency of ING4, BECN1 and PIK3C3 siRNAs. After the silencing cells were treated with complete medium (Untreated), Torin1 (300nM) or Rapamycin (1 μM) for 6h in presence of BAFA1 (1 μM) for the last 3h. Data are means ± SD (*n* = 5). Statistical analysis was performed by Student's t test in comparison with the control condition (***p < 0.001) or in comparison with the Torin1 (### *p* < 0.001) or Rapamycin ($ *p* < 0.05, $$$ *p* < 0.001) conditions. **D.** Representative photomicrographs of ING4 knockdown (siING4A) U2OS cells stably expressing GFP-LC3B treated with complete medium (CM) or 3 methyladenine (3MA; 10mM) or wortmannin (WM; 10 μM) for 6h in presence of BAFA1 (1 μM) for the last 3h. (**E**) Quantification of the number of GFP-LC3B dots in ING4 knockdown (siING4A) U2OS cells, treated with chemical inhibitors of PIK3C3: 3MA or WM for 6h in presence of BAFA1 (1 μM) for the last 3h. Data are means ± SD (*n* = 5). Statistical analysis was performed by Student's t test in comparison with the CM of siUNR condition (***p < 0.001) or in comparison with the CM of siING4A condition (### *p* < 0.001).

Inhibition of the lipid kinase activity of PIK3C3 by ING4. Autophagy is tied to the BECN1-dependent activation of PIK3C3, resulting in the generation of PI3P that can be visualized by means of RFP-tagged short peptide (FYVE) that specifically binds to PI3P-containing subcellular vesicles [43]. Depletion of ING4 with three siRNAs induced the redistribution of RFP-FYVE from a diffuse to a punctiform pattern, indicating activation of PIK3C3 in baseline or nutrient free conditions (Figure 6A-6B). Moreover, knockdown of BECN1 or PIK3C3 (Figure 6C), or its pharmacological inhibition by 3-methyladenine (3MA) or wortmannin (WM) abolished the induction of GFP-LC3 puncta in response to ING4 depletion (siING4A) (Figure 6D-6E). Altogether, these results indicate that ING4 inhibition causes autophagy through the activation of the lipid kinase activity of the BECN1/PIK3C3 complex.

Concluding remarks. The results reported in this paper support the contention that ING4 is indeed constitutively interacting with BECN1 in mammalian cells that are cultured in nutrient-rich conditions. However, upon nutrient depletion the BECN1 complex alters its composition, in line with the reported plasticity of the BECN1 interactome [8], and ING4 dissociates from BECN1. Similar dissociation processes have been reported for B-cell lymphoma 2 (BCL-2) [9], B-cell lymphoma-extra large (Bcl-xL) [44], TGFβ-activated kinase 1 (TAK1)-binding proteins 2 and 3 (TAB2 and TAB3) [12], Golgi associated pathogenesis related-1 (GAPR1) [45] that all appear to be sufficient to cause BECN1/VPS34 inhibition because knockdown of BCL-2, Bcl-xL, TAB2, TAB3 and GAPR1 is sufficient to stimulate autophagy. Moreover, small molecules or peptides designed to competitively disrupt the interaction of BECN1 and BCL-2, Bcl-xL, TAB2, TAB3 and GAPR1 induce the formation of GFP-LC3B puncta, [9, 12, 44, 45] further supporting the pleiotropic nature of the suppressive interactions affecting the BECN1 complex. The mechanisms leading to ING4 dissociation from BECN1 in conditions of starvation are elusive. Previous work revealed that nutrient depletion causes the deacetylation of multiple autophagy-regulatory proteins [46] including that of BECN1, which is required for autophagy induction [47]. At this stage, it remain to be determined whether ING4 can affect BECN1 acetylation or whether BECN1 acetylation influences the binding of ING4. Irrespective of these unknowns, it appears that the dissociation of the ING4-BECN1 interaction correlates with the induction of autophagy by other stimuli than starvation including addition of torin1, a specific inhibitor of mTOR (not shown).

It appears intriguing that ING4, which is generally viewed as a transcription-regulatory factor interacting with methylated histones (in particular H3K4me3) [48] and DNA [49] has major cytoplasmic functions as well. However, ING4 reportedly acts on NF-κB as an E3 ubiquitin ligase [36], and at least a fraction of ING4 is located in the cytoplasm [24], supporting the possibility that it exerts important extranuclear functions. Previous work has established that TP53 tonically inhibits autophagy by an interaction with RB1-inducible coiled-coil protein 1 (RB1CC1) [50–52] and other mechanisms [53]. Another case is provided by the transcription factor STAT3 that can inhibit autophagy in the cytoplasm by inhibiting eukaryotic translation initiation factor 2-alpha kinase 2 (EIF2AK2) [54]. Hence, ING4 apparently constitutes yet another example of multifunctional proteins that act at several subcellular localizations, namely, as nuclear (co)transcription factors and as direct inhibitors of the autophagic machinery.

Altogether, it appears that ING4 constitutes yet another regulator of autophagy that acts on the BECN1 complex, repressing its PIK3C3-mediated lipid kinase activity. Future work must determine whether and to which extent this function contributes to the tumor suppressive action of ING4.

## MATERIALS AND METHODS

### Chemicals, cell lines and culture conditions

Unless otherwise indicated, chemicals were purchased by Sigma-Aldrich (St Louis, USA), and media and supplements for cell culture from Gibco-Invitrogen (Carlsbad, USA). Rapamycin was obtained by Tocris Bioscience (Ellisville, USA). All cells were maintained in standard culture conditions (37°C, 5% CO_2_). Human osteosarcoma U2OS cells (and their GFP-LC3 and FYVE-RFP-expressing derivatives) were cultured in Dulbecco's modified Eagle's medium (DMEM) supplemented with 10% Fetal bovine serum (FBS) and 10 mM HEPES. Human colon carcinoma HCT116 WT and GFP-LC3 stable expressing cells were maintained in McCoy's medium supplemented with 10% FCS, 100 mg/l sodium pyruvate and 10 mM HEPES. For serum and nutrient starvation, cells were cultured in serum-free Earle's balanced salt solution (EBSS) for 6 h.

### siRNAs, plasmids and transfections

Cells at 50% confluence were transfected with a custom-made, non-targeting siRNA (siUNR, 5′-GCCGGUAUGCCGGUUAAGUdTdT-3′) or with siRNAs specific to PIK3C3 (5′-ACG- GTGATGAATCATCTCCdTdT-3′), BECN1 (5′-UUCCGUAAGGAACAAGUCGG- d TdT-3′), ING4A (5′-rgrgrCrCrArCUrgrArgUrAUrAUrgrArgU TT-3′), ING4B (5′-rgrCrgrCrArCrArArgUrCrCUrgrArgUrAU TT-3′) or ING4C (Scbt, Sc-60850). siRNA transfections were performed by means of the Lipofectamine® RNAiMAX reagent (Invitrogen), according to the manufacturer's instructions.

Plasmid transfection was carried out by means of the FuGENE® HD Transfection Reagent (Promega), as recommended by the manufacturer. A plasmid encoding a His-BECN1 was co-transfected with the empty vector pcDNA3.1 (Invitrogen) or with mammalian expression vectors encoding Flag-ING4.

### Immunoblotting and immunoprecipitation

Immunoblotting was performed following standard procedures. In short, 20*μ*g of protein were separated on NuPAGE Novex Bis-Tris 4-12% pre-cast gels (Invitrogen-Life Technologies, Carlsbad, CA, USA) and transferred to Immobilon polyvinylidene difluoride membranes (Merck-Millipore, Darmstadt, Germany). Unspecific binding was reduced by incubating the membranes for 1h in 0.05% Tween 20 (v/v in Tris-buffered saline, TBS) supplemented with 5% w/v bovine serum albumin (Euromedex, Souffelweyersheim, France). Following, membranes were probed with antibodies specific for LC3B (CST, #2275), SQSTM1 (Abnova, #H00008878-M01), ING4 (Invitrogen, #40-7700) beta Actin (ab20272), Flag (Sigma, #F3040), His (CST, #2365) and Acetylated lysine (CST, #9441), BECN1 (Scbt, sc-11427), PIK3C3 (CST, #4263). Primary antibodies were revealed with species-specific immunoglobulin G conjugated to horseradish peroxidase (Southern Biotech, Birmingham, AL, USA), followed by chemiluminescence analysis with the SuperSignal West Pico reagent by means of an ImageQuant 4000 (GE Healthcare, Little Chalfont, UK). A primary antibody that specifically recognizes actin was used to ensure equal lane loading. Western blot images were quantified by using ImageJ software.

For immunoprecipitation, 7×10^6^ cells were lysed as previously described [55], and 400 μg of proteins was pre-cleared for 1 h with 30 μl of Pure Proteome™ Protein G Magnetic Beads (Millipore), followed by incubation for 4 h in the presence of 2 μg of specific antibodies or IgG controls. Subsequent immunoblotting was carried out using TrueBlot™-HRP (eBioscience, San Diego, USA) secondary antibodies.

### High-throughput assessment of LC3 lipidation and FYVE dots

Five x 10^3^ U2OS FYVE-RFP, U2OS or HCT116 cells stably expressing LC3-GFP were seeded into black 96-well μclear imaging plates (Greiner Bio-One) and allowed to adapt for 24 h. Thereafter the cells were treated with nutrient-free medium, 3-methyladenine, wortmannin (with or without bafilomycin A1) and respective controls and incubated for additional 6h before fixation in 3.7 % (w/v) paraformaldehyde (PFA) in phosphate-buffered saline (PBS) supplemented with 1 μM Hoechst 33342 at 4°C over night. Upon fixation, PFA was substituted with PBS and a minimum of four view fields per well were acquired by means of an ImageXpress micro XL automated bioimager (Molecular Devices) equipped with a PlanApo 20X/0.75 NA objective (Nikon).

### Data processing and statistical analyses

Unless otherwise specified, independent experiments were performed in triplicate parallel instances and repeated three times. Microscopy images were segmented and analyzed by means of the MetaXpress (Molecular Devices) software. Unless otherwise specified, data are presented as means ± S.D. Significance was assessed by means of two-tailed Student's t-test.

GK is supported by the Ligue contre le Cancer (équipe labelisée); Agence National de la Recherche (ANR) - Projets blancs; ANR under the frame of E-Rare-2, the ERA-Net for Research on Rare Diseases; Association pour la recherche sur le cancer (ARC); Cancéropôle Ile-de-France; Institut National du Cancer (INCa); Institut Universitaire de France; Fondation pour la Recherche Médicale (FRM); the European Commission (ArtForce); the European Research Council (ERC); the LeDucq Foundation; the LabEx Immuno-Oncology; the RHU Torino Lumière, the SIRIC Stratified Oncology Cell DNA Repair and Tumor Immune Elimination (SOCRATE); the SIRIC Cancer Research and Personalized Medicine (CARPEM); and the Paris Alliance of Cancer Research Institutes (PACRI).

## SUPPLEMENTARY MATERIALS FIGURES


