# Supplementary material for: Inhibitor of growth protein 4 interacts with Beclin 1 and represses autophagy
Source: Oncotarget. 2017 Jul 6;8(52):89527–38. doi: 10.18632/oncotarget.19033 (PMC5685689; doi:10.18632/oncotarget.19033)
Supplement: Supplementary file 1 [file oncotarget-08-89527-s001.pdf]

Inhibitor of growth protein 4 interacts with Beclin 1 and represses autophagy

SUPPLEMENTARY MATERIALS

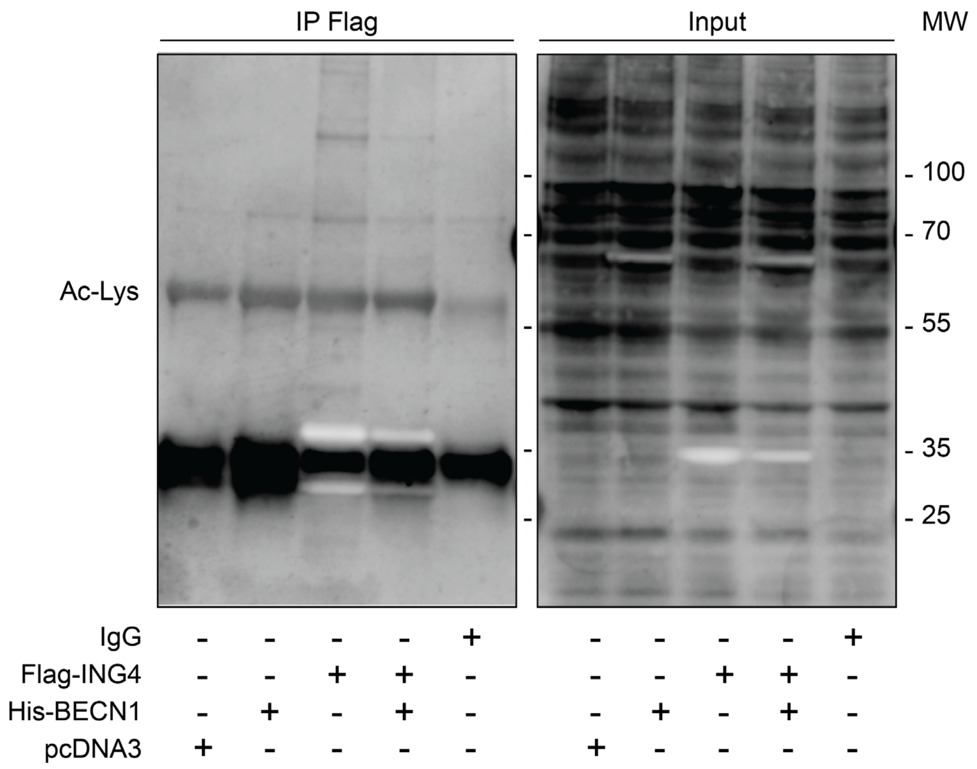

Supplementary Figure 1: ING4 overexpression is not responsible of Beclin1 acetylation. The indicated constructs, namely Flag-tagged ING4 (Flag-ING4) and His-tagged BECN1 (His-BECN1) were transfected into U2OS cells alone or in combination. The pcDNA3 construct was transfected as internal control of the experiment. Forty-eight hours later, ING4 was immunoprecipitated with a specific antibody for Flag and the precipitate was separated by SDS-PAGE and revealed with an antibody specific for Acetylated lysine (Ac-Lys). Results are representative of three independent experiments. Immunoprecipitation (IP); molecular weight (MW).

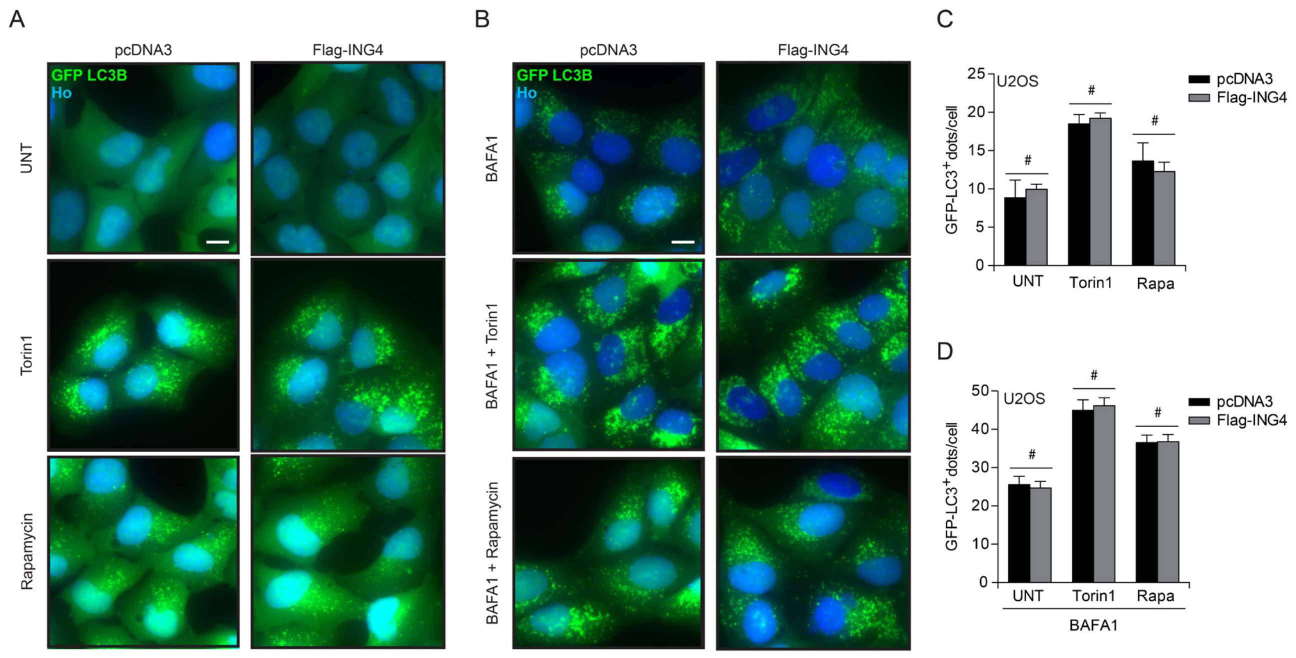

Supplementary Figure 2: Overexpression of ING4 has not inhibitory effect on autophagy induction in U2OS cells. (A, B) Representative photomicrographs of empty vector pcDNA3 (pcDNA3) and ING4 (Flag-ING4) overexpressing U2OS cells stably expressing GFP-LC3B, treated in absence (untreated, UNT) or presence of Torin1 (300nM) or Rapamycin (1μM) for 6h, in absence (A) or in presence (B) of Bafilomycin A1 (BAFA1, 1 μM) for the last 3h. Hoechst 33342 (Ho, blue) represents nuclear staining. GFP-LC3B puncta (green) correspond to autophagosomes. Scale bars: 10 μm. (C) Quantification of GFP-LC3B puncta in cells treated as in (A). (D) Quantification of GFP-LC3B puncta in cells treated as in (B). Data are means ± SD (n=5). Statistical analysis was performed by Student's t test in comparison as indicated, # p>0.05, not significant. Results are representative of three independent experiments.
